# Zinc and iron dynamics in human islet amyloid polypeptide-induced diabetes mouse model

**DOI:** 10.1038/s41598-023-30498-y

**Published:** 2023-03-15

**Authors:** Ayako Fukunaka, Mari Shimura, Takayuki Ichinose, Ofejiro B. Pereye, Yuko Nakagawa, Yasuko Tamura, Wakana Mizutani, Ryota Inoue, Takato Inoue, Yuto Tanaka, Takashi Sato, Tatsuya Saitoh, Toshiyuki Fukada, Yuya Nishida, Takeshi Miyatsuka, Jun Shirakawa, Hirotaka Watada, Satoshi Matsuyama, Yoshio Fujitani

**Affiliations:** 1 Laboratory of Developmental Biology and Metabolism, Institute for Molecularand Cellular Regulation, Gunma University, Maebashi, Gunma Japan; 2 Department of Refractory Viral Infection, Research Institute, National Center for Global Health and Medicine, Tokyo, Japan; 3 RIKEN SPring-8 Center, Kouto, Hyogo Japan; 4 Inorganic Analysis Laboratories, Toray Research Center, Inc., Otsu, Shiga Japan; 5 Laboratory of Diabetes and Metabolic Disorders, Institute for Molecular and Cellular Regulation, Gunma University, Maebashi, Gunma Japan; 6 Department of Materials Physics, Nagoya University, Nagoya, Japan; 7 Graduate School of Engineering, Osaka University, Osaka, Japan; 8 Laboratory of Bio-Response Regulation, Graduate School of Pharmaceutical Sciences, Osaka University, Osaka, Japan; 9 Faculty of Pharmaceutical Sciences, Tokushima Bunri University, Tokushima, Japan; 10 Department of Metabolism and Endocrinology, Juntendo University Graduate School of Medicine, Tokyo, Japan; 11 Department of Endocrinology, Diabetes, and Metabolism, Kitasato University Hospital, Sagamihara, Kanagawa Japan; 12 Center for Therapeutic Innovations in Diabetes, Juntendo University Graduate School of Medicine, Tokyo, Japan

**Keywords:** Endocrine system and metabolic diseases, Metabolic disorders

## Abstract

Metal homeostasis is tightly regulated in cells and organisms, and its disturbance is frequently observed in some diseases such as neurodegenerative diseases and metabolic disorders. Previous studies suggest that zinc and iron are necessary for the normal functions of pancreatic β cells. However, the distribution of elements in normal conditions and the pathophysiological significance of dysregulated elements in the islet in diabetic conditions have remained unclear. In this study, to investigate the dynamics of elements in the pancreatic islets of a diabetic mouse model expressing human islet amyloid polypeptide (hIAPP): hIAPP transgenic (hIAPP-Tg) mice, we performed imaging analysis of elements using synchrotron scanning X-ray fluorescence microscopy and quantitative analysis of elements using inductively coupled plasma mass spectrometry. We found that in the islets, zinc significantly decreased in the early stage of diabetes, while iron gradually decreased concurrently with the increase in blood glucose levels of hIAPP-Tg mice. Notably, when zinc and/or iron were decreased in the islets of hIAPP-Tg mice, dysregulation of glucose-stimulated mitochondrial respiration was observed. Our findings may contribute to clarifying the roles of zinc and iron in islet functions under pathophysiological diabetic conditions.

## Introduction

Approximately a third of the human proteome contains metal cations, either in the form of cofactors with catalytic functions or as structural support. To guarantee the proper maintenance of the homeostasis of these metals, cells and organisms have evolved highly sophisticated machinery involved in the transport, storage, and distribution of metals^1^. Studies have also shown that the imbalance of these metal ions in some tissues is closely associated with the onset and/or progression of various diseases. For example, patients with chronic hepatitis C frequently demonstrate iron overload in the serum and liver. In Alzheimer’s disease patients, imbalance of metal ions in the brain are frequently observed and are thought to be associated with Aβ deposition and tau hyperphosphorylation, a hallmark of Alzheimer’s disease^2,3^.

Pancreatic islets consist of 5 types of endocrine cells, including insulin producing β cells. Pancreatic β cells comprise approximately 80% of the cells in the islets and are known to contain very high concentrations of zinc compared with other islet cells^4^. In particular, insulin secretory granules have been shown to have a high zinc content and are packed along with islet amyloid polypeptide (IAPP) within β cells^5^. Iron is also indispensable for β cells, considering the importance of mitochondrial function in β cells. It has been shown that iron is important for normal glucose-stimulated insulin secretion; however, excess iron causes oxidative stress and increases apoptosis in β cells^6,7^. Thus, zinc and iron play essential roles in pancreatic β-cell biology, and the dysfunction of their homeostasis has been reported to be implicated in the pathogenesis of type 2 diabetes^4,6^.

Whereas most previous studies on the basic pathogenic mechanism of diabetes have used mouse models, there are many differences between human and mouse diabetes. For instance, the structure and function of pancreatic islets are different between mouse models and humans^8^. One of the most intriguing differences between mouse diabetes models and human patients is that the deposition of islet amyloid is found in more than 90% of human diabetes patients, but not in mouse models. This is owing to differences in their amino acid residues of IAPP (also known as amylin) between them^9^. Human and mouse IAPP share similar amino acid sequences in their N- and C-terminal regions, whereas their amino acids in the middle region substantially differ. Rodent IAPP has a proline substitution in the middle region, and hence does not form a β-sheet structure, and thus lacks the ability to self-oligomerize IAPP and form amyloid. To investigate the role of human IAPP (hIAPP), several mouse models have been developed, such as transgenic hIAPP overexpression models, and hIAPP knock-in mice^10–13^. These models clearly demonstrated that the expression of hIAPP induces toxic effects on β cells, likely owing to apoptosis and amyloidogenesis, although these mice models do not exhibit obesity. However, the molecular mechanisms by which hIAPP exerts cytotoxic effects have not yet been clarified.

In this study, we have investigated the dynamics of elements of islets derived from hIAPP transgenic (hIAPP-Tg) mice. We applied synchrotron X-ray scanning fluorescence microscopy (SXFM) and inductively coupled plasma mass spectrometry (ICP-MS) for imaging and quantitative analysis of elements, respectively. SXFM enables the mapping of multiple intracellular elements at the sub-organelle level by the combination of a synchrotron radiation source and a sub-100-nm X-ray beam focusing system^14–20^, and ICP-MS can quantify the concentrations of multiple elements with high sensitivity and precision (Fig. S1). Notably, zinc in the islets of hIAPP-Tg mice was decreased in the early stage of diabetes, whereas iron was reduced with the progression of hyperglycemia. We discuss the possible association between the decrease in these 2 essential elements and the progression of diabetes based on the hIAPP expression.

## Results

### hIAPP-Tg mice demonstrate aging-dependent hyperglycemia and amyloid deposition in their islets

**Figure 1:**
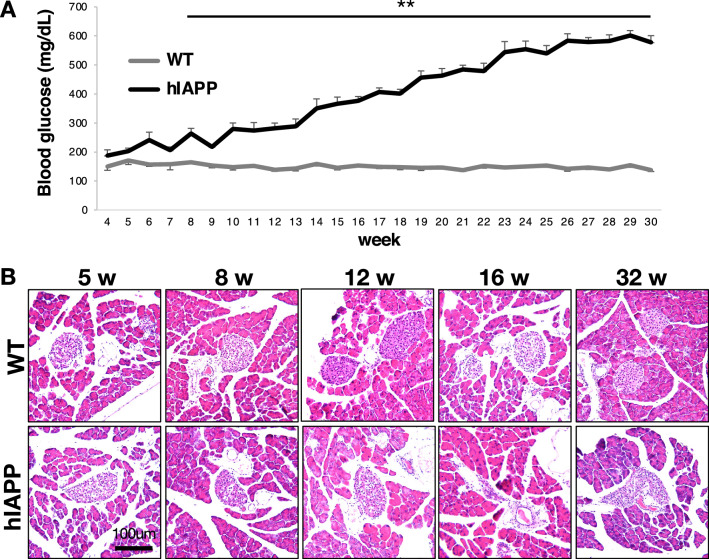
Phenotype of hIAPP-Tg mice. (**A**) Non-fasting blood glucose levels of WT mice (n = 3–11) and hIAPP-Tg mice (n = 4–9). (**B**) HE staining of pancreas sections from 5-, 8-, 12-, 16-, and 32-week-old hIAPP-Tg mice and WT mice. Data are shown as the mean ± SEM. **p < 0.01 (WT vs. hIAPP). Scale bar, 100 μm.

To analyze the phenotype of hIAPP-Tg mice, blood glucose measurements and hematoxylin & eosin (HE) staining were performed using 5, 8, 12, 16, and 32-week-old in wild type (WT) and hIAPP-Tg mice. Non-fasting blood glucose levels were slightly increased in 5-week-old hIAPP-Tg mice compared with littermate WT mice, although the difference was not significant. hIAPP-Tg mice showed a significant increase in blood glucose level after 8-week-old (Fig. 1A). The islets of hIAPP-Tg mice appeared to have almost the same morphology as the islets of WT mice until 12 weeks of age. However, after 12 weeks, some islets of hIAPP-Tg mice became smaller and irregular in shape (Fig. 1B). Furthermore, insulin signals gradually decreased in hIAPP-Tg mice with aging (Fig. S2A), which is consistent with previous report^10,21–23^. Notably, a slightly eosinophilic appearance, which is the typical staining pattern of amyloid, was observed in 32-week-old hIAPP-Tg mice (Fig. S2B, arrows). The accumulation of amyloid was also detected in 32-week-old hIAPP-Tg mice by Thioflavin-T staining, which is a classical procedure to detect amyloid (Fig. S2B and C, arrows). These data confirm age-dependent hyperglycemia and deposition of islet amyloid in hIAPP-Tg mice, as previously reported^10,11,13,21–23^.

### Iron and zinc dynamically decreased in the islets of aged hIAPP-Tg mice, a phenotype associated with diabetes

**Figure 2:**
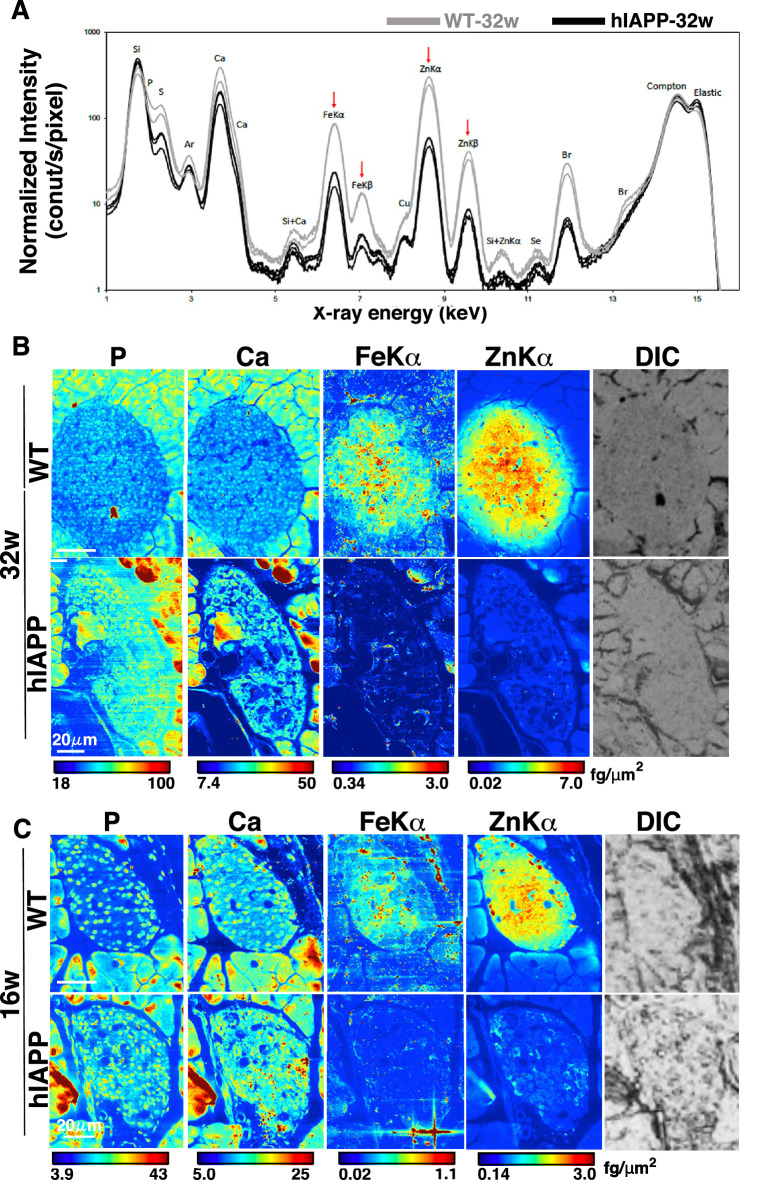
X-ray fluorescence images of islets from 16- and 32-week-old WT and hIAPP-Tg mice. (**A**) X-ray fluorescence spectra of islet sections from 32-week-old mice. Arrows indicate peak signals of FeKα, FeKβ, ZnKα, and ZnKβ x-ray emission lines. Measurement was performed three times for each section. Gray line: a spectrum for a section from control mouse; black line: a spectrum for a section from hIAPP. X-ray energy, 15 keV; beam size, 500 $\times$ 500 nm. (**B**) Representative mapping images of (**A**). (**C**) Mapping images of islets from indicated 16-week-old mice. WT: WT mice, hIAPP: hIAPP-Tg mice, Color bar, femtograms per square micrometer; DIC: differential interference contrast image; Scale bar, 20 μm.

We performed SXFM imaging to observe the distribution of elements in the pancreatic islets. X-ray fluorescence energy spectrum showed that iron (X-ray emission lines: FeKα or FeKβ), zinc (ZnKα or ZnKβ), and bromine were substantially decreased in the islets of 32-week-old hIAPP-Tg mice compared with WT mice (black-colored lines, Fig. 2A), while all Compton and Elastic scattering X-rays, which influenced by the thickness of a sample basement, were quite repeatable (Fig. 2A). Bromine is abundantly contained in animal foods and bedding; however, even WT mice in our study showed variable bromine concentrations in their pancreatic tissue, resulting that bromine decrease shown in Fig. 2A was not repeated (Fig. S3A). We hence concluded that the differences in bromine were caused by individual differences. We focused on zinc and iron, as they are essential elements. Their mapping data showed a significant reduction at FeKα and ZnKα in the entire region of islets, whereas phosphorus and calcium mapping were comparable to those of WT mice (Fig. 2B). Another independent experiment using islets of 32-week-old hIAPP-Tg mice showed similar results, demonstrating the reproducibility of the results (Fig. S3). Similar results were also obtained from the islets of 16-week-old hIAPP-Tg mice (Fig. 2C), suggesting that the decrease in zinc and iron probably starts before 16 weeks of age in hIAPP-Tg mice (Fig. 2). Quantitative analysis of islets by ICP-MS was not possible because it is difficult to isolate islets from the pancreatic tissues hIAPP-Tg mice more than 16 week of age, for an unknown reason.

### Zinc in hIAPP-Tg mouse islets decreased in the early stage of diabetes, while iron gradually decreased with the progression of hyperglycemia

**Figure 3:**
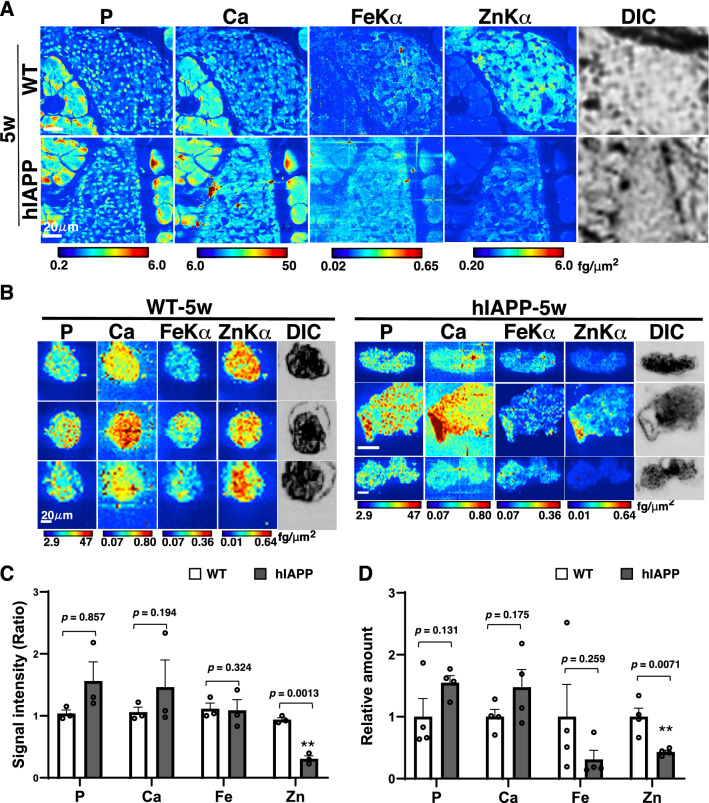
Decreased zinc in islets of 5-week-old hIAPP-Tg mice. (**A**) Mapping images of pancreatic islets from 5-week-old mice. (**B**) Mapping images of isolated islets from 5-week-old mice. Color bar, femtograms per square micrometer; DIC: differential interference contrast image; Scale bar, 20 μm. (**C**) Quantification of signal intensities in (**B**). (**D**) Relative amounts of each element in isolated islets were measured using ICP-MS (n = 4 for each genotype). WT: WT mice, hIAPP: hIAPP-Tg mice, Data are shown as means ± SEM. **p < 0.01 (WT vs. hIAPP).

To clarify whether zinc or iron dynamics were affected by the development of diabetes, we first analyzed 5-week-old hIAPP-Tg mice and found that their blood glucose levels were comparable to those of WT mice (Fig. 1A). We performed SXFM imaging on pancreatic islet tissues (Fig. 3A) and isolated islets from pancreatic tissues (Fig. 3B). Both mapping data suggested that zinc signals in the islets tended to be decreased in hIAPP-Tg mice, whereas iron signals was comparable to that of WT mice. The signal intensities of the mapping from isolated islets also suggested that zinc was significantly decreased in hIAPP-Tg mice (*p* = 0.0013), whereas iron was not (*p* = 0.324, Fig. 3C). To confirm these results quantitatively, we performed ICP-MS using isolated islets from pancreatic tissues. The results from the ICP-MS analysis supported the mapping data (Fig. 3D), suggesting that iron in the islets of hIAPP-Tg mice did not change, although zinc significantly decreased in the early stage of diabetes.

**Figure 4:**
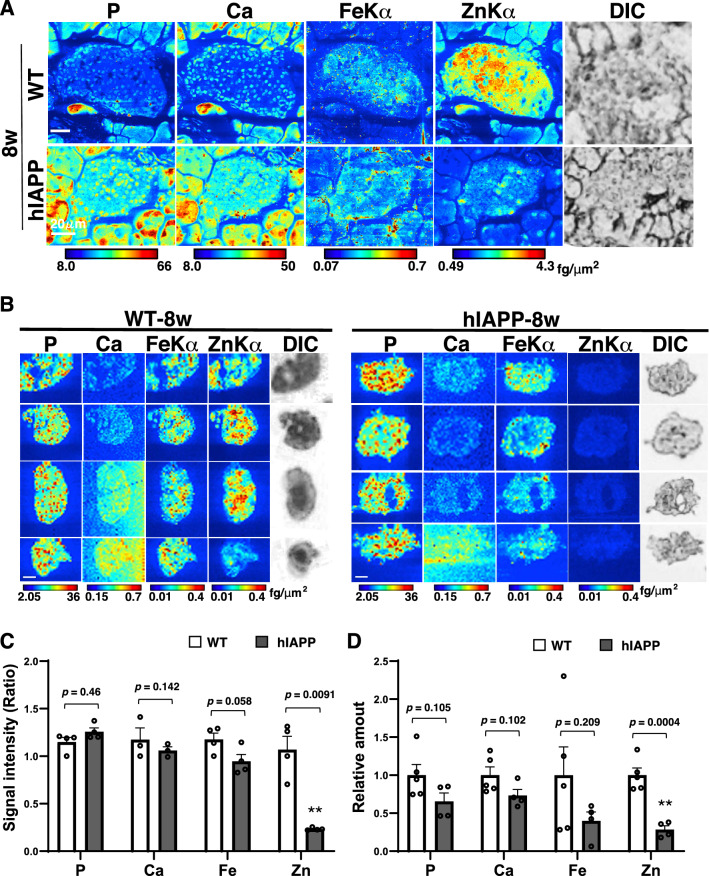
Iron level in islets is partially decreased in 8-week-old hIAPP-Tg mice. (**A**) Mapping images of pancreatic islets from 8-week-old mice. (**B**) Mapping images of isolated islets from 8-week-old mice. Color bar, femtograms per square micrometer; DIC: differential interference contrast image; Scale bar, 20 µm. (**C**) Quantification of signal intensities in (**B**). (**D**) Relative amounts of each element in isolated islets were measured using ICP-MS (n = 5,4 for each genotype). WT: WT mice, hIAPP: hIAPP-Tg mice, Data are shown as means ± SEM. **p < 0.01 (WT vs. hIAPP).

We next analyzed using pancreatic tissue and isolated islets of hIAPP-Tg mice and WT mice at 8 weeks of age, when blood glucose levels had started to be significantly increased without any obvious weight changes (Figs. 1A and S4). Mapping data from pancreatic tissue (Fig. 4A) and isolated islets from pancreatic tissues (Fig. 4B) of hIAPP-Tg mice showed that zinc decreased significantly (*p* = 0.0091), and iron showed a decreasing tendency (*p* = 0.058) in the islets of hIAPP-Tg mice (Fig. 4A–C). ICP-MS showed that zinc decreased significantly (*p* = 0.0004), whereas iron did not (*p* = 0.209) in the islets of hIAPP-Tg mice (Fig. 4D). These data suggest that zinc remains obviously decreased, while iron did not much change in the islets of 8-week-old hIAPP-Tg mice.

**Figure 5:**
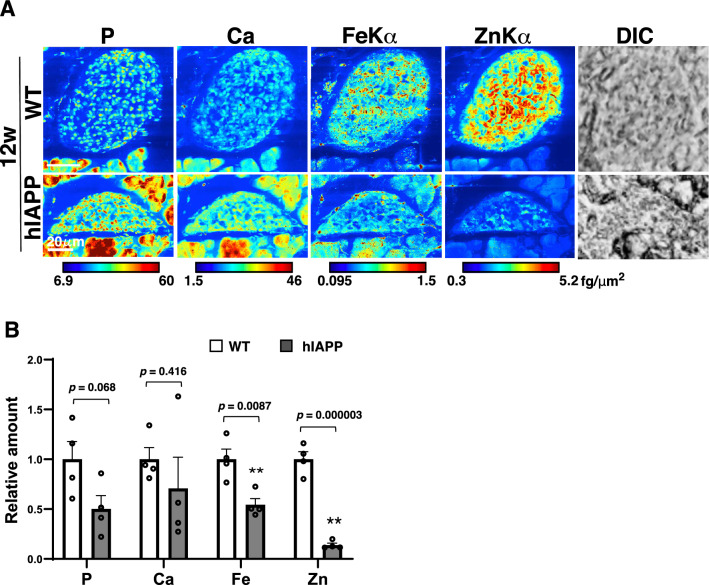
Decreased iron in the islets of 12-week-old hIAPP-Tg mice. (**A**) Mapping images of pancreatic islets from 12-week-old mice. Color bar, femtograms per square micrometer; DIC: differential interference contrast image; Scale bar, 20 µm. (**B**) Relative amounts of each element in isolated islets were measured using ICP-MS (n = 4 for genotype). WT: WT mice, hIAPP: hIAPP-Tg mice, Data are given as means ± SEM. ** *p* < 0.01 (WT vs. hIAPP).

We next analyzed 12-week-old hIAPP-Tg mice, in which significant blood glucose level changes were observed without any obvious weight changes (Figs. 1A and S4). We found a slight decrease in iron in the core region of 12-week-old hIAPP-Tg mouse islets, where β cells are localized^8^. On the other hand, zinc remained low in the entire islets (Fig. 5A). Data from ICP-MS showed that iron (*p* = 0.0087) and zinc (*p* = 0.0013) decreased significantly in the islets of hIAPP-Tg mice in diabetic conditions (Fig. 5B). Taken together, these data suggest that the decrease in zinc reflected a prediabetic or the early stages of diabetes. In contrast, iron in the islets of hIAPP-Tg mice gradually decreased concurrently with the development of diabetic phenotypes after 8–12 weeks of age.

### The possible association between metal contents and cellular functions

**Figure 6:**
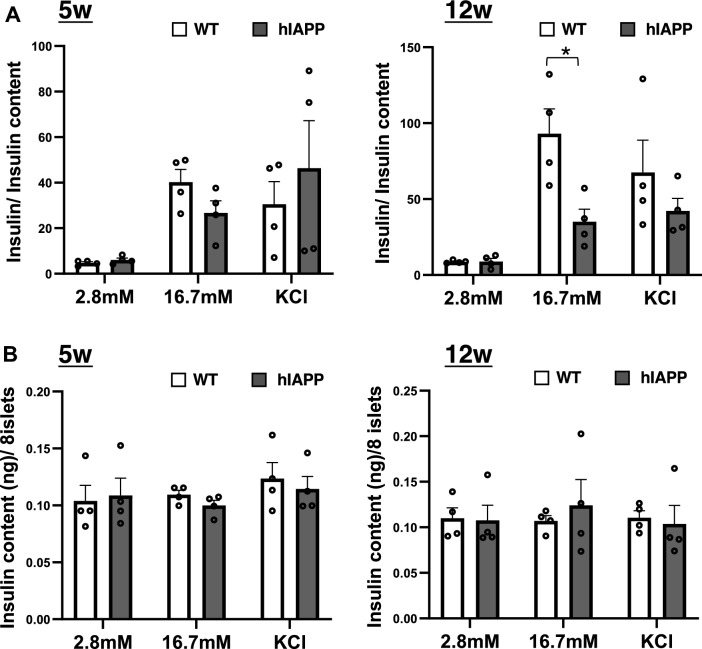
Glucose-stimulated insulin secretion (GSIS) is decreased in 12-week-old hIAPP-Tg mice. (**A**) GSIS in 5 and 12-week-old indicated islets. Islets were incubated in KRB buffer containing 2.8 or 16.7 mM glucose, or 40 mM KCl for 60 min (n = 4 per group). (**B**) Insulin content in 5 and 12-week-old mice islets (n = 4 per group). WT: WT mice, hIAPP: hIAPP-Tg mice, Data are shown as means ± SEM. *p < 0.05 (WT vs. hIAPP).

**Figure 7:**
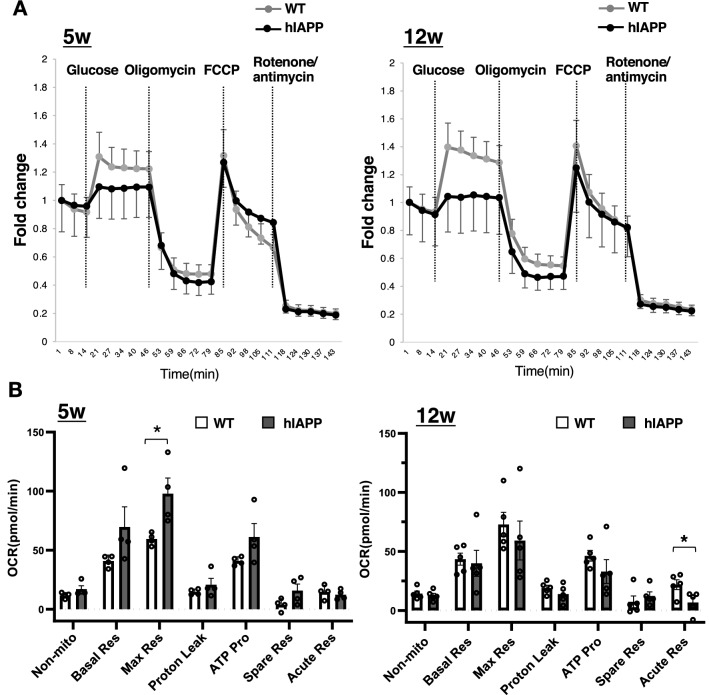
Mitochondrial function of 5- and 12-week-old hIAPP-Tg mice. (**A**) Fold-increase in the oxygen consumption rate of mice (n = 4 for 5-week-old mice, n = 5 for 12-week-old mice). (**B**) Analyzed data are shown as a graph. Non-mito: nonmitochondrial oxygen consumption; Basal Res: basal respiration, Max Res: maximal respiration, Proton Leak, ATP Pro: ATP production, Spare Res: spare respiratory capacity, Acute Res: acute response. Max Res and Acute Res are calculated by the equation as (maximum rate measurement after FCCP injection)—(non-mitochondrial respiration) and (late rate measurement before oligomycin injection)—(last rate measurement before acute injection), respectively. WT: WT mice, hIAPP: hIAPP-Tg mice, Data are shown as means ± SEM. *p < 0.05 (WT vs. hIAPP).

To investigate the possible association between blood glucose levels and zinc and iron, we investigated the glucose-stimulated insulin secretion (GSIS) in pancreatic islet cells. GSIS from isolated islets was significantly reduced in 12-week-old hIAPP-Tg mice (Fig. 6A and B, right panel), while GSIS was not significantly changed in 5-week-old hIAPP-Tg mice compared to WT mice (Fig. 6A and B, left panel). Given that there was no difference in KCl-induced insulin secretion between the two groups, the deficiency of signaling events upstream of KATP channel closure appears to be responsible for the defects in GSIS. Considering the fact that the mitochondrion is a key machinery involved in the regulation of GSIS in β cells and studies have shown that disruption in zinc and iron homeostasis may seriously affect mitochondrial function, leading to an impaired energy state and susceptibility to disease development^24,25^, we investigated mitochondrial function in pancreatic islets cells. The analysis of mitochondrial respiration using a flux analyzer demonstrated that glucose-stimulated mitochondrial respiration (Acute Res) was significantly suppressed in the islets of 12-week-old hIAPP-Tg mice compared with WT mice (Fig. 7A and B, right panels). In contrast, there was no change in Acute Res of 5-week-old hIAPP-Tg mice when compared to WT mice (Fig. 7A and B, left panels). Maximal respiration (Max Res) induced by the addition of the uncoupler FCCP was upregulated in 5-week-old hIAPP-Tg mice, which might be due to the upregulation of compensation machinery for hIAPP toxicity (Fig. 7B, left panel). These results suggest that changes in both iron and/or zinc dynamics in pancreatic islets may affect Acute Res in the islets.

## Discussion

Previous studies have suggested that relatively high concentrations of zinc and iron were necessary for the normal function of pancreatic β cells^4,7,26,27^. Our data showed that a decrease in zinc and iron is associated with hyperglycemia in the islets of hIAPP-Tg mice, implicating an association between decreased zinc and/or iron in islets and the onset of human diabetes.

### Decrease in zinc in islets

Notably, imaging and quantitative analysis of metals in islets showed a decrease in zinc in the pancreatic islets of hIAPP-Tg mice in the early stage of diabetes. The question then arises as to whether the decrease in zinc is due to the expression of hIAPP. To clarify that, we transiently expressed hIAPP in the rat insulinoma INS-1 cell line and analyzed zinc and iron contents in the cells (Fig. S5). Zinc content in INS-1 cells expressing hIAPP was comparable to that in control cells, suggesting that the decreased zinc content was not due to only hIAPP expression. A decrease in zinc may require a long-term expression of hIAPP or/and its associated environmental conditions such as inflammation in vivo^28^. Notably, a significant decrease in zinc content in pancreatic tissues from various genetic mouse models of type 2 diabetes in the early stage of the disease, such as *db/db* mice (having a mutation in the leptin receptor) and *ob/ob* mice (having a mutation in the leptin gene) have been reported^29,30^, suggesting that the decrease in zinc is not dependent only on hIAPP expression. A recent study reported that the treatment of β cells from the islets of WT mice with inflammatory cytokines caused a significant reduction in zinc^31^. Therefore, a decrease in zinc may be associated with chronic inflammation prior to the onset of diabetes.

### Decrease in iron in islets

It has been reported that iron is increased in patients with type 2 diabetes, and therapeutic phlebotomy, a procedure that is used to reduce iron levels, was found to improve β-cell function in patients with pathological iron overload^32,33^. These findings suggest that there is an imbalance of iron metabolism in diabetic patients. However, the actual iron concentration within the islets was not reported. We observed that iron content in islets gradually decreased concurrently with the development of diabetic phenotype in hIAPP-Tg mice. Recently, mice lacking iron regulatory protein 2; a regulator of cellular iron homeostasis were reported to have a decreased iron content in β cells, and developed diabetes^6^, which is consistent with our result that a decrease in iron occurs concurrently with the onset of diabetes, although the mechanism of the decrease islet iron content remains unclear to date.

### A decrease in zinc and/or iron is associated with the dysfunction of mitochondria in β cells

It is important to understand how the decrease in zinc and/or iron alters the function of islets. Cellular iron deficiency results in reduced activity of Fe-S cluster-based complexes in the mitochondria, which is associated with impaired mitochondrial respiration^34^, and the restoration of intracellular iron levels can reverse these effects^34,35^. Zinc has been reported to restore impaired mitochondrial pyruvate transport, oxidative phosphorylation, and ultimate energy metabolism^36^. Our data showed an alteration in glucose-stimulated mitochondrial respiration following zinc and/or iron was reduced in hIAPP-Tg mice older than 12 weeks of age. This is consistent with a previous study which reported similar alterations in glucose-stimulated mitochondrial respiration in rats transgenic for hIAPP (HIP rats); an alteration associated with activation of the HIF1α/PFKFB3 signaling pathway leading to the disengagement of glycolysis from the mitochondrial TCA cycle^24,37^. The authors of this study suggested that this adaptive metabolic response induces β-cell dysfunction accompanied by a deficient response to glucose stimulation^24^. Notably, our data revealed that 12-week-old of hIAPP-Tg mice showed iron deficiency in islets, which might trigger the activation of the HIF1α signaling pathway and lead to increased glycolysis^38^.

### Study limitations and future perspectives

It remains unclear whether a decrease in zinc and/or iron in islets is the result of or the cause of diabetes. However, it is possible that a decrease in zinc and/or iron worsens the situation. In our study, hIAPP-Tg mice show a decrease in iron and zinc at 12 weeks old, and dysregulation in glucose-stimulated mitochondrial respiration that might lead to the dysfunction of mitochondrial function in β cells. As this study presents, we only identified the association between metal dynamics and the progressive dysregulation of islets, further analysis is needed to resolve the link between metal levels and islet functions using hIAPP-Tg mice or islets from diabetes patients. A combination of metal analysis with other omics studies will provide clarifications on the pathophysiological relevance of iron and zinc dynamics in diabetes^39^.

The interaction of metal ions with hIAPP may also affect its structure, causing the formation of misfolded IAPP, which can undergo oligomer and amyloid formation^40,41^. There are several in vitro studies reporting on the effects of metal ions on the formation of soluble hIAPP oligomers and mature amyloid^42–47^; however, there are limited information on this issue regarding the physiological conditions in studies using mouse models or human samples. SXFM will become a useful tool to visualize the interaction between metals and hIAPP-derived amyloid formation. Further analysis is needed in the future to clarify the association between metals and hIAPP amyloid formation.

## Methods

### Mouse experiments

All mice were housed in specific pathogen-free barrier facilities, maintained under a 12-h light/dark cycle, given water ad libitum, and fed with standard rodent chow (Oriental Yeast, Tokyo, Japan). Blood glucose levels were measured using a glucose analyzer (Glutest Mint, Sanwa Chemical Co., Nagoya, Japan). hIAPP-Tg mice were obtained from Jackson Laboratories (Strain No. 008232) and mice were backcrossed with C57BL/6 J mice for more than seven generations. Mice were euthanized by anesthesia with isoflurane.

### Pancreatic islet culture

Mouse islet isolation was performed as described previously^4,12,48^. Isolated islets were cultured in RPMI 1640 medium supplemented with 10% fetal bovine serum (FBS) and 1% penicillin/streptomycin.

### GSIS

GSIS from mouse islets was investigated as described previously^48^. Briefly, eight size-matched islets were incubated in HRKB buffer containing glucose or KCl for 60 min. Insulin concentration of supernatants of isolated islets was analyzed by mouse insulin enzyme-linked immunosorbent assay kit (Morinaga Institute of Biological Science, Yokohama, Japan). Insulin secretion data were corrected by the insulin content of the islets.

### SXFM

Differential interference contrast images of each tissue section were obtained prior to SXFM measurements using an Olympus DP22 microscope. SXFM was performed using the undulator beamline BL29XU at the SPring-8 synchrotron radiation facility by combining a Kirkpatrick-Baez type X-ray focusing system, xy scanning stage for scanning sample mounting, and an energy dispersive X-ray detector (Vortex-90EX; Hitachi High-Technologies Science America, Inc., Northridge, CA, USA)^15^. Monochromatic X-rays at 15 keV were focused down to 500 × 500 nm^2^ for a large-area scan. A typical photon flux for the 500-nm beam is approximately 2 × 10^11^ photons/s. X-ray fluorescence spectra were recorded using a 1-s exposure for each pixel. When the region of interest was relatively wide, it was separated into several parts for the measurement. The fluorescence signals of each element of interest were extracted and normalized based on the incident beam intensity. After scanning the whole area, the distributions of various elements were visualized digitally. SXFM produced superimposed signals from the samples in the vertical direction. In addition to the mapping images, the element concentration per area (µm^2^) was analyzed quantitatively using thin nickel and platinum films, the thickness and density of which were decided in advance. Signal intensities per interested region in the TIFF images were acquired using ImageJ software (National Institutes of Health, Bethesda, MD, USA).

### ICP-MS

For isolated islet samples of small amounts (from 5, 8, and 12-week-old-mice), 0.5 mL of nitric acid solution in a PFA jar (ARAM Co., Osaka, Japan) was used. The jar was heated at 100 °C for 20 min using an ETHOS 1 microwave oven (Milestone Srl, Sorisole, Italy). Concentrations of Ca, Fe, P, and Zn were determined using ICP-MS (ELEMENT XR, Thermo Fisher Scientific Inc, Bremen, Germany) with resolution R-10,000 for ^44^Ca, and R-4,000 for ^31^P, ^56^Fe, and ^66^Zn. The INS-1 cells were digested with 0.5 mL of nitric acid (Tamapure-AA-100, Tama Chemical Co. Ltd., Kanagawa, Japan) at 180 °C for 20 min in an ETHOS 1 microwave oven (Milestone Srl, Sorisole, Italy) and then diluted with ultrapure water (manufactured by PURELAB Option-R 7 and PURELAB flex UV, Veolia Water Solutions and Technologies, Paris, France) to a 5-mL volume. Concentrations of P, Ca, Fe, and Zn were determined using ICP-MS (ELEMENT XR, Thermo Fisher Scientific Inc., Bremen, Germany) with resolution R-10,000 for ^44^Ca, and R-4,000 for ^31^P, ^56^Fe, and ^66^Zn.

### Mitochondrial respiration in islets

Mitochondrial respiration in mouse islets was measured as previously described^49^. Briefly, isolated islets (20 islets/well) from hIAPP-Tg mice and WT mice were incubated in RPMI 1640 medium containing 5.6 mM glucose, 1 mM pyruvate, and 10% FBS. The islets were washed with PBS and seeded into poly-L-lysine coated XF96 cell culture microplates (Agilent Technologies, Palo Alto, CA). The culture microplates were then centrifuged at 500 rpm for 7 min at room temperature and incubated for 1 to 2 h at 37 °C in a non-CO_2_ incubator. XF RPMI Medium (Agilent Technologies) containing 5.6 mM glucose, 1 mmol/L pyruvate, and 2 mM L-glutamine was used as the assay medium. The oxygen consumption rate and extracellular acidification rate were measured using a Seahorse SX96 Analyzer (Agilent Technologies). Islets were sequentially exposed to glucose (11.1 mM), oligomycin (4 µM), carbonyl cyanide 4-phenylhydrazone (FCCP) (10 µM), and rotenone/antimycin A (2.5 µM). Wave 2.6.0 software (Agilent Technologies) was used to analyze non-mitochondrial oxygen consumption, basal respiration, maximal respiration, proton leak, ATP production, spare respiratory capacity, and acute response. Non-mitochondrial oxygen consumption is calculated by the equation as minimum rate measurement after rotenone/antimycin A injection. Basal respiration is calculated by the equation as (last rate measurement before the first injection)—(non-mitochondrial respiration rate). Maximal respiration is calculated by the equation as (maximum rate measurement after FCCP injection)—(non-mitochondrial respiration). Proton leak is calculated by the equation as (minimum rate measurement after oligomycin injection)—(non-mitochondrial respiration). ATP production is calculated by the equation as (last rate measurement before oligomycin injection)—(minimum rate measurement after oligomycin injection). Spare respiratory capacity is calculated by the equation as (maximal respiration)—(basal respiration). Acute response is calculated by the equation as (last rate measurement before oligomycin injection)—(last rate measurement before acute injection).

### Statistical analysis

All quantitative data were reported as the mean ± SEM. Student *t*-test was performed for comparison between groups. Welch’s t-test was performed in the experiments of Figs. 3C, 4C. *p* < 0.05 was considered a significant difference between control an experimental group.

### Ethical approval

All experimental animal care was performed in accordance with institutional and national guidelines and regulations. The study protocol was approved by the Institutional Animal Care and Use Committee of Gunma University (Permit #19–025). The study is reported in accordance with ARRIVE guidelines.

## Supplementary Information

Supplementary Information 1.

Supplementary Information 2.

Supplementary Information 3.

## Data Availability

The datasets used and/or analyzed during this study are included in this published article and its supplemental information files.

## Abbreviations

SXFM: Scanning X-ray fluorescence microscopy
ICP-MS: Inductively coupled plasma mass spectrometry
IAPP: Islet amyloid polypeptide
hIAPP-Tg: Human IAPP transgenic
HE: Hematoxylin & eosin
OCR: Oxygen consumption rate
T2D: Type2 diabetes
