# Supplementary material for: Zinc and iron dynamics in human islet amyloid polypeptide-induced diabetes mouse model
Source: Sci Rep. 2023 Mar 15;13:3484. doi: 10.1038/s41598-023-30498-y (PMC10017767; doi:10.1038/s41598-023-30498-y)
Supplement: Supplementary file 1 — Supplementary Information 1. [file 41598_2023_30498_MOESM1_ESM.pdf]

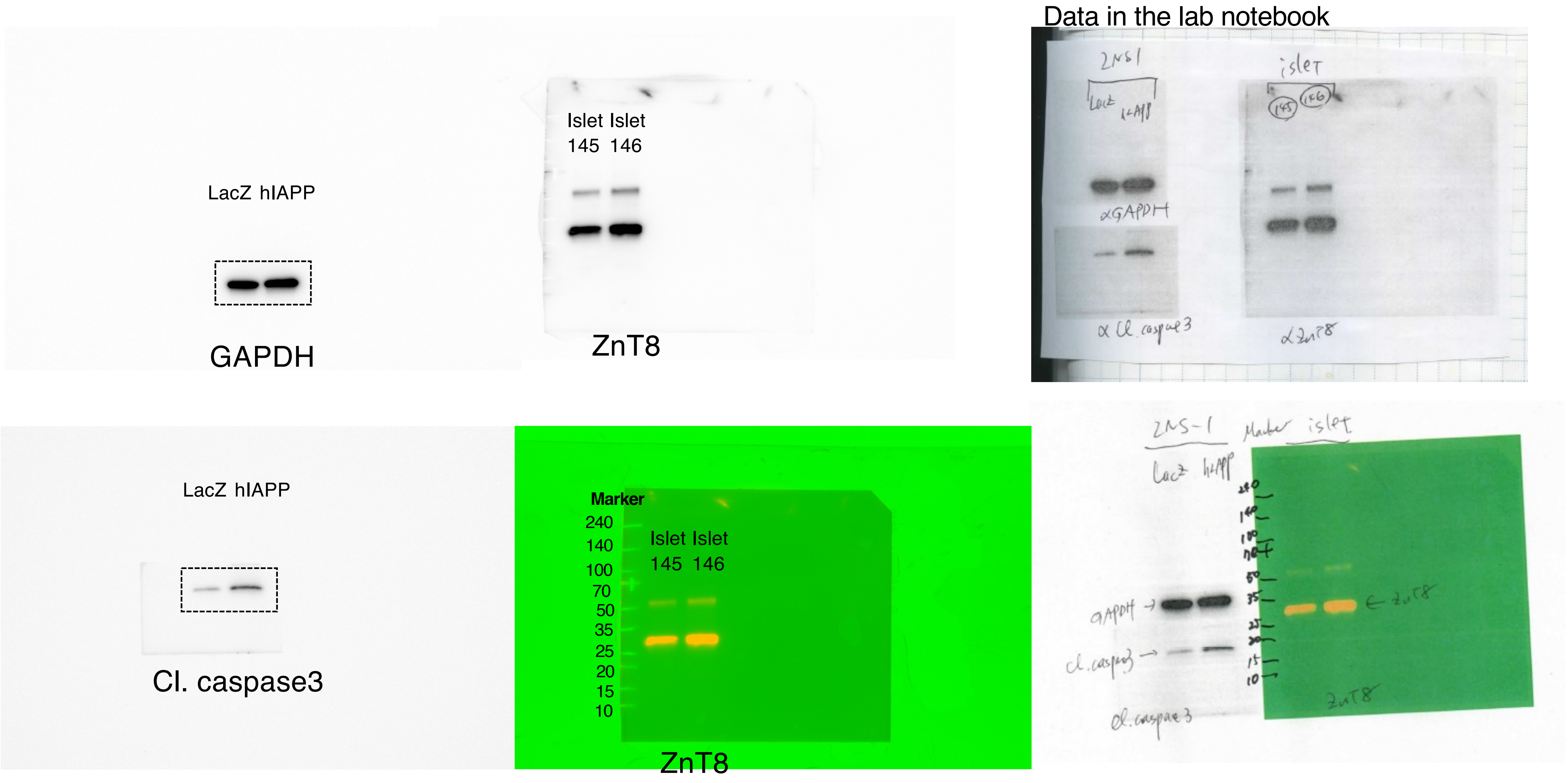
Data in the lab notebook
LacZ hIAPP
GAPDH
Islet Islet
145 146
ZnT8
LacZ hIAPP
Cl. caspase3
Marker
240
140
100
70
50
35
25
20
15
10
Islet Islet
145 146
ZnT8

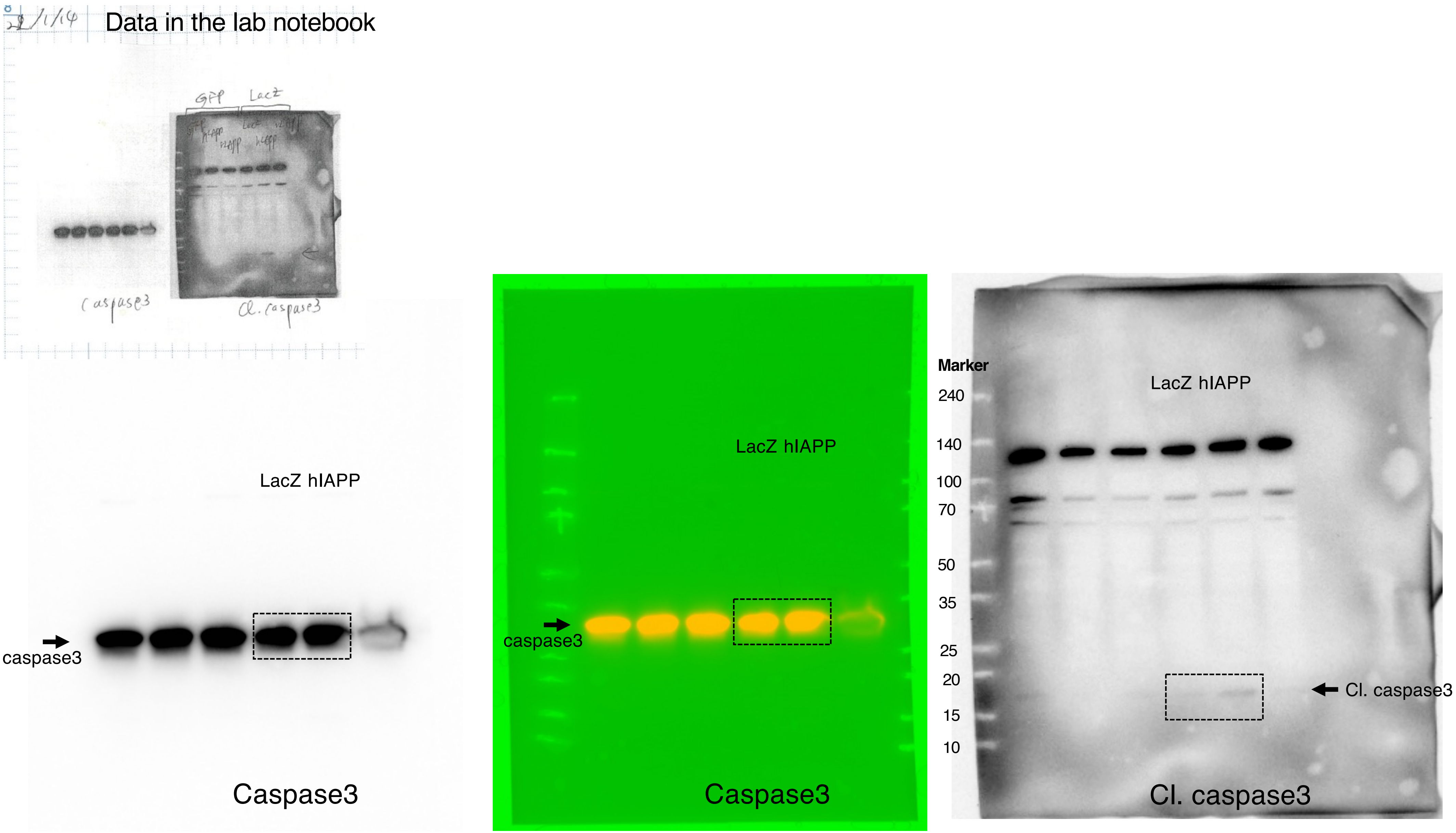
Data in the lab notebook
GFP
LacZ
caspase3
Cl. caspase3
LacZ hIAPP
caspase3
Caspase3
LacZ hIAPP
caspase3
Caspase3
Marker
240
140
100
70
50
35
25
20
15
10
LacZ hIAPP
Cl. caspase3
Cl. caspase3

GAPDH antibody
CST
Catalog #2118
Data sheet

https://www.cellsignal.com/products/primary-antibodies/gapdh-14c10-rabbit-mab/2118

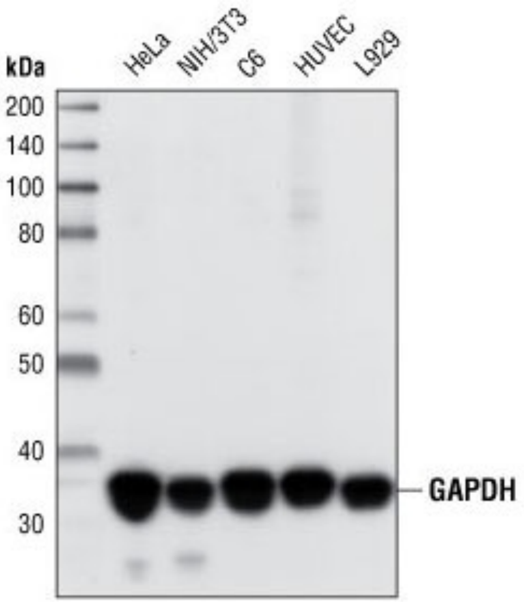

Molecular weight 35kDa

Cl. Caspase3 antibody
CST antibody
Catalog #9664
Data sheet

https://www.cellsignal.jp/products/primary-antibodies/cleaved-caspase-3-asp175-5a1e-rabbit-mab/9664

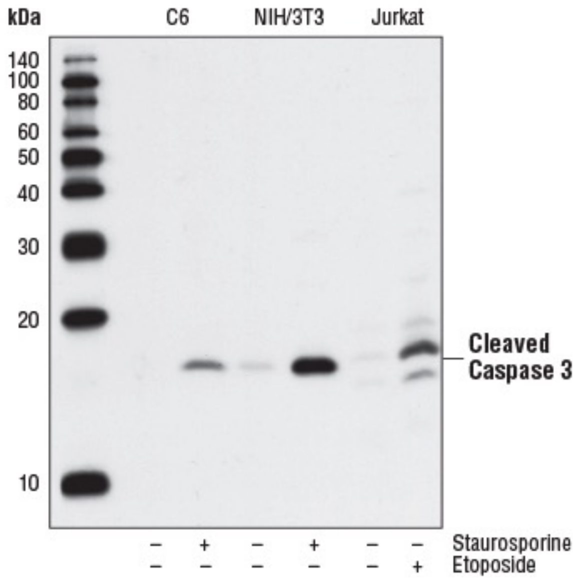

Molecular weight 17,19kDa

Caspase3 antibody
CST antibody
Catalog #9662
Data sheet

https://www.cellsignal.jp/products/primary-antibodies/caspase-3-antibody/9662

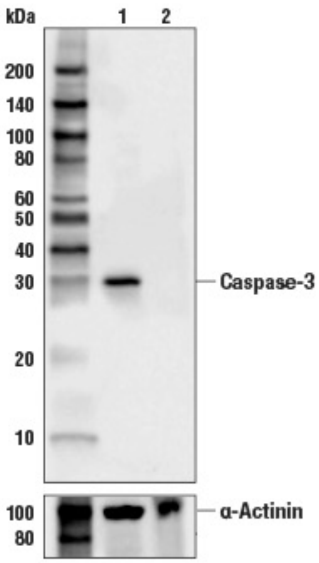

Molecular weight 30kDa
