# Supplementary material for: Zinc and iron dynamics in human islet amyloid polypeptide-induced diabetes mouse model
Source: Sci Rep. 2023 Mar 15;13:3484. doi: 10.1038/s41598-023-30498-y (PMC10017767; doi:10.1038/s41598-023-30498-y)
Supplement: Supplementary file 2 — Supplementary Information 2. [file 41598_2023_30498_MOESM2_ESM.docx]

**Supporting Information**

**Supplemental figure legends**

**Fig. S1. Schematic representation of the study.** SXFM and ICP-MS analyses were used to screen for changes in element contents in the islets of hIAPP-Tg mice that occur with the progression of diabetes. Then, the association between metals and cellular function in the islets of hIAPP-Tg mice was analyzed.

**Fig. S2.** **Insulin staining of WT and hIAPP-Tg mice and amyloids deposits in 32-week-old hIAPP mice.** (A) Insulin staining of pancreas sections from 5-, 8-, 12-, 16-, and 32-week-old hIAPP-Tg mice and WT mice. (B) HE staining of a pancreas section from a 32-week-old hIAPP-Tg mouse. (C) Thioflavin T staining of a pancreas section from a 32-week-old hIAPP-Tg mouse independent from (B). Arrows indicate amyloid. Scale bar, 100 μm

**Fig. S3. X-ray fluorescence images of islet sections from 32-week-old WT and hIAPP-Tg mice.** (A) X-ray fluorescence spectra of islet sections from 32-week-old mice in different mice from Fig. 2B. Arrows indicate peak signals of FeKα, FeKβ, ZnKα, and ZnKβ x-ray emission lines. X-ray energy, 15 keV. Measurement was performed three times for each section. Gray line: a spectrum for a section from control mouse; black line: a spectrum for a section from hIAPP. (B) Representative image of (A). Beam size, 1,500 $\times1,$500 nm. Scale bar, 20 μm. White square, a region taken for higher resolution in (C). (C) Higher resolution images of (A). Beam size, 500 $\times$500 nm. Scale bar, 6.7 μm. WT: WT mice, hIAPP: hIAPP-Tg mice, Color bar, femtograms per square micrometer; DIC: differential interference contrast image.

**Fig. S4. Body weights and glucose levels of hIAPP-Tg mice used for ICP-MS.** (A) Body weights of 5-, 8-, and 12-week-old mice. (B) Blood glucose levels of 5-, 8-, and 12-week-old mice. WT: WT mice, hIAPP: hIAPP-Tg mice, WT mice (n = 4-5), hIAPP-Tg mice (n = 4), Data are shown as the mean ± SEM. ***p* < 0.01 (WT vs hIAPP).

**Fig. S5. Expression of hIAPP in INS-1 cells did not result in significant changes in metal contents.** (A) Schematic representation of the experimental design. INS-1 cells were infected with adenoviruses expressing LacZ and hIAPP and metal contents were measured by ICP-MS after 72 h of adenovirus infection. (B) The number of cells not stained with Trypan Blue was counted. (C) Western blotting of the indicated proteins in INS-1 cells after the infection of an adenovirus expressing LacZ (Ctrl) or hIAPP. (D) Relative amounts of each element in indicated cells were measured using ICP-MS. Data are shown as means ± SEM. **p* < 0.05, ***p* < 0.01(Ctrl vs hIAPP).

**Supplemental Methods**

**hIAPP adenovirus infection**

Recombinant adenoviruses expressing LacZ and hIAPP were generated as described previously ^1^. An adenovirus expressing only LacZ was used as a control. High titer adenovirus (> 10^8^ infectious units per mL) was obtained by repeated infection into HEK293 cells and purified using Virakit (Virapure, San Diego, CA, USA).

**Immunoblotting**

Immunoblotting was performed as described previously ^2^ ^3^. The blotted membrane was blocked with a blocking solution (5% skim milk and 0.1% Tween-20 in tris-buffered saline) and then incubated with an anti-Cl. caspase 3 antibodies (1:1,000; Cell Signaling Technology, Danvers, MA, USA) or an anti-GAPDH antibody (1:1,000; Cell Signaling Technology) in a blocking solution. Immobilon Western Chemiluminescent HRP Substrates (Millipore, Burlington, MA, USA) were used for detection. Fluoroscopic images were obtained using a LAS3000 plus image analyzer (Fujifilm, Tokyo, Japan).

**Immunohistochemistry**

After anesthetization, mice were perfused intracardially with 4% paraformaldehyde and fixed as described previously for immunohistochemical staining of tissues using anti-insulin antibody (1:50, Dako-Agilent, Santa Clara, CA) ^1^.

References

1 Shigihara, N. *et al.* Human IAPP-induced pancreatic β cell toxicity and its regulation by autophagy. *J Clin Invest* **124**, 3634-3644, doi:10.1172/jci69866 (2014).

2 Fukunaka, A. *et al.* Tissue nonspecific alkaline phosphatase is activated via a two-step mechanism by zinc transport complexes in the early secretory pathway. *J Biol Chem* **286**, 16363-16373, doi:10.1074/jbc.M111.227173 (2011).

3 Fukunaka, A. *et al.* Zinc transporter ZIP13 suppresses beige adipocyte biogenesis and energy expenditure by regulating C/EBP-β expression. *PLoS Genet* **13**, e1006950, doi:10.1371/journal.pgen.1006950 (2017).
