# Supplementary material for: Zinc and iron dynamics in human islet amyloid polypeptide-induced diabetes mouse model
Source: Sci Rep. 2023 Mar 15;13:3484. doi: 10.1038/s41598-023-30498-y (PMC10017767; doi:10.1038/s41598-023-30498-y)
Supplement: Supplementary file 3 — Supplementary Information 3. [file 41598_2023_30498_MOESM3_ESM.pdf]

Fig. S1

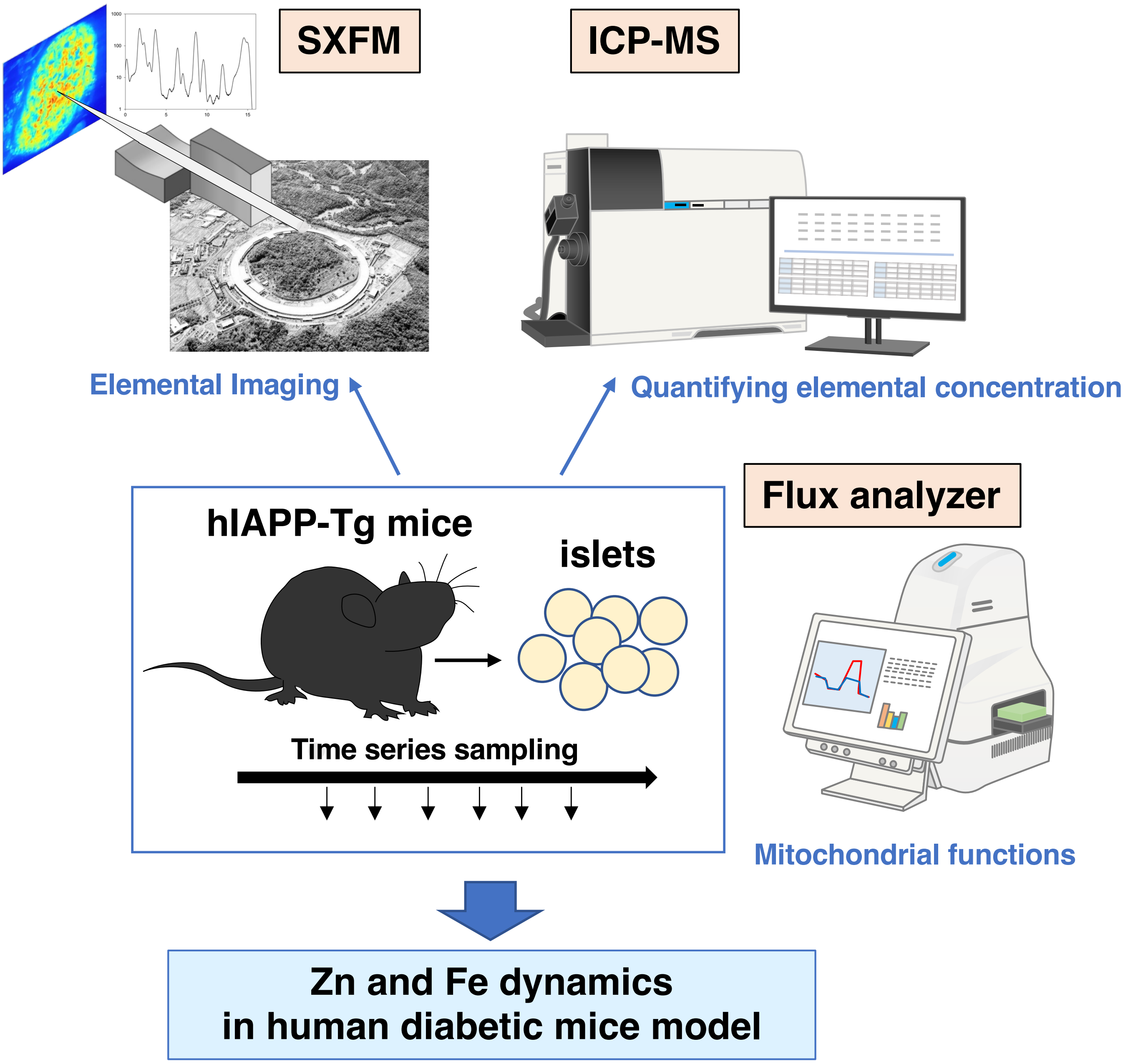

Fig. S1. Schematic representation of the study.
SXFM and ICP-MS analyses were used to screen for changes in element contents in the islets of hIAPP-Tg mice that occur with the progression of diabetes. Then, the association between metals and cellular function in the islets of hIAPP-Tg mice was analyzed.

Fig. S2

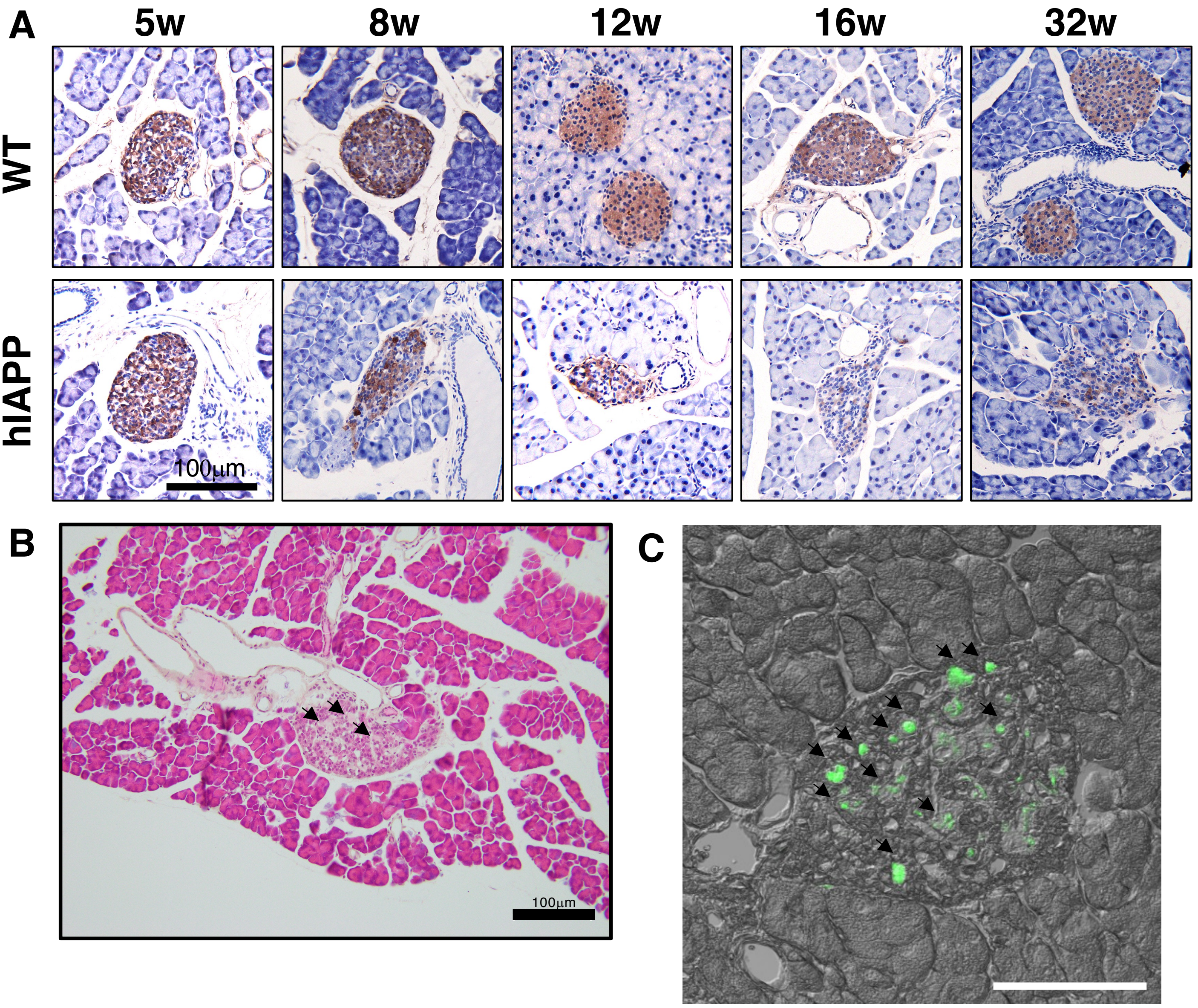

Fig. S2. Insulin staining of WT and hIAPP-Tg mice and amyloids deposits in 32-week-old hIAPP mice.

(A) Insulin staining of pancreas sections from 5-, 8-, 12-, 16-, and 32-week-old hIAPP-Tg mice and WT mice. (B) HE staining of a pancreas section from a 32-week-old hIAPP-Tg mouse. (C) Thioflavin T staining of a pancreas section from a 32-week-old hIAPP-Tg mouse independent from (B). Arrows indicate amyloid. Scale bar, 100 µm

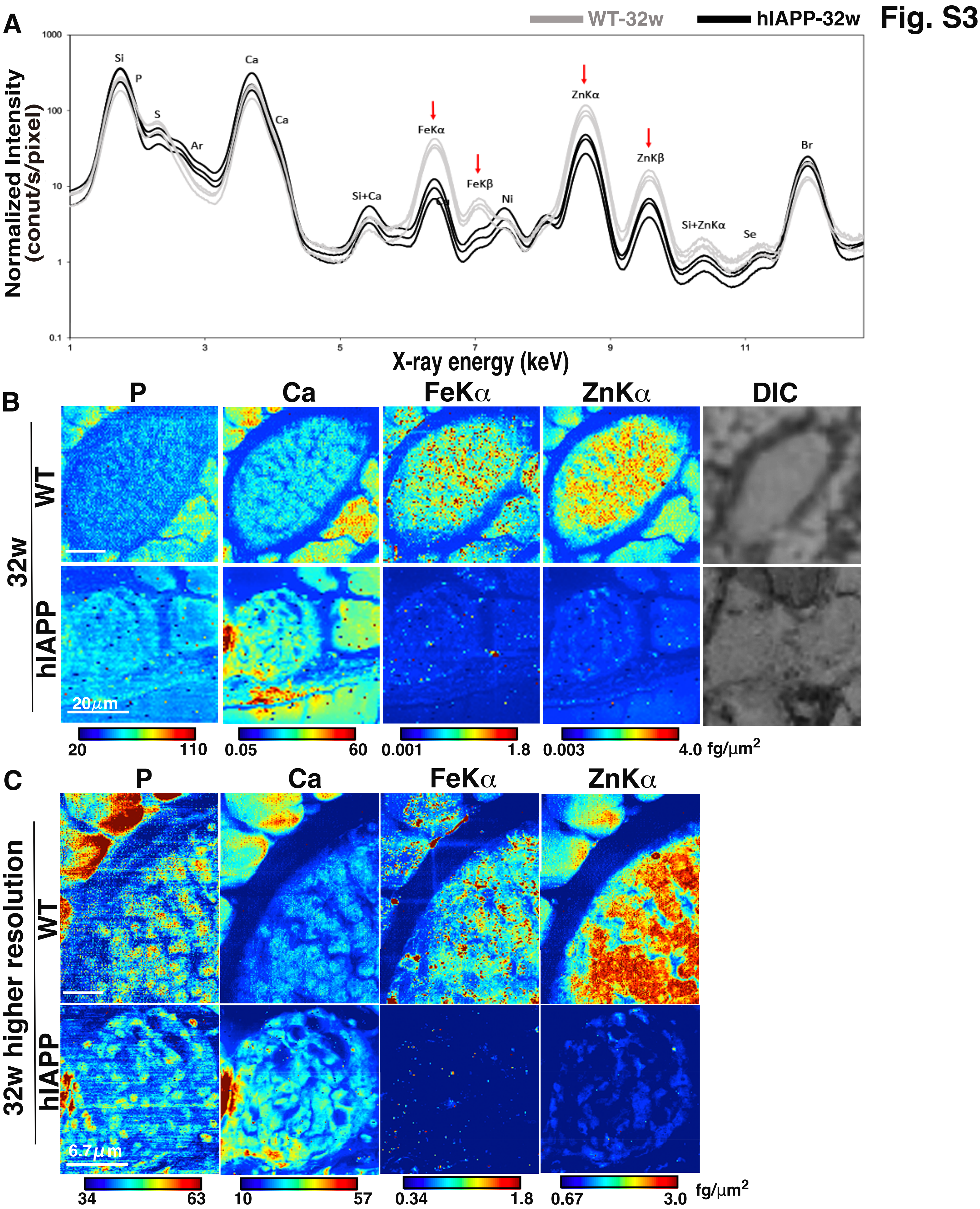

Fig. S3. X-ray fluorescence images of islet sections from 32-week-old WT and hIAPP-Tg mice. (A) X-ray fluorescence spectra of islet sections from 32-week-old mice in different mice from Fig. 2B. Arrows indicate peak signals of FeKα, FeKβ, ZnKα, and ZnKβ x-ray emission lines. X-ray energy, 15 keV. Measurement was performed three times for each section. Gray line: a spectrum for a section from control mouse; black line: a spectrum for a section from hIAPP. (B) Representative image of (A). Beam size, 1,500 × 1,500 nm. Scale bar, 20 μm. White square, a region taken for higher resolution in (C). (C) Higher resolution images of (A). Beam size, 500 × 500 nm. Scale bar, 6.7 μm. WT: WT mice, hIAPP: hIAPP-Tg mice, Color bar, femtograms per square micrometer; DIC: differential interference contrast image; Scale bar, 20 μm

Fig. S4

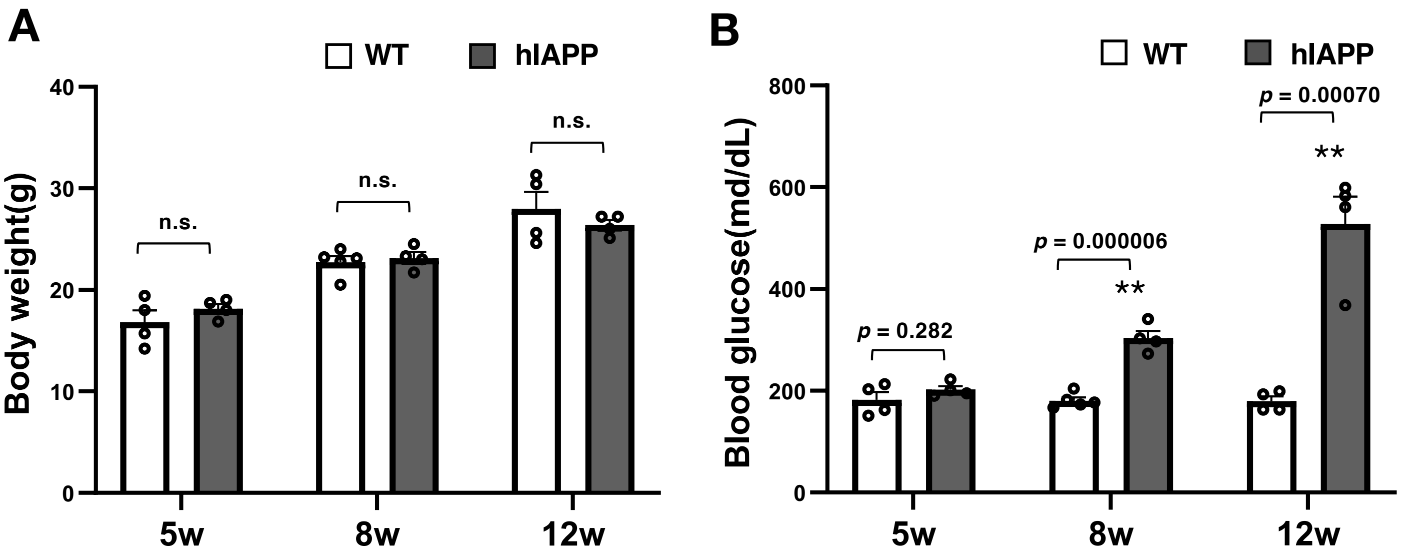

Fig. S4. Body weights and glucose levels of hIAPP-Tg mice used for ICP-MS.
(A) Body weights of 5-, 8-, and 12-week-old mice. (B) Blood glucose levels of 5-, 8-, and 12-week-old mice. WT: WT mice, hIAPP: hIAPP-Tg mice, WT mice (n = 4-5), hIAPP-Tg mice (n = 4), Data are shown as the mean ± SEM. **$p<0.01$ (WT vs hIAPP)

Fig. S5

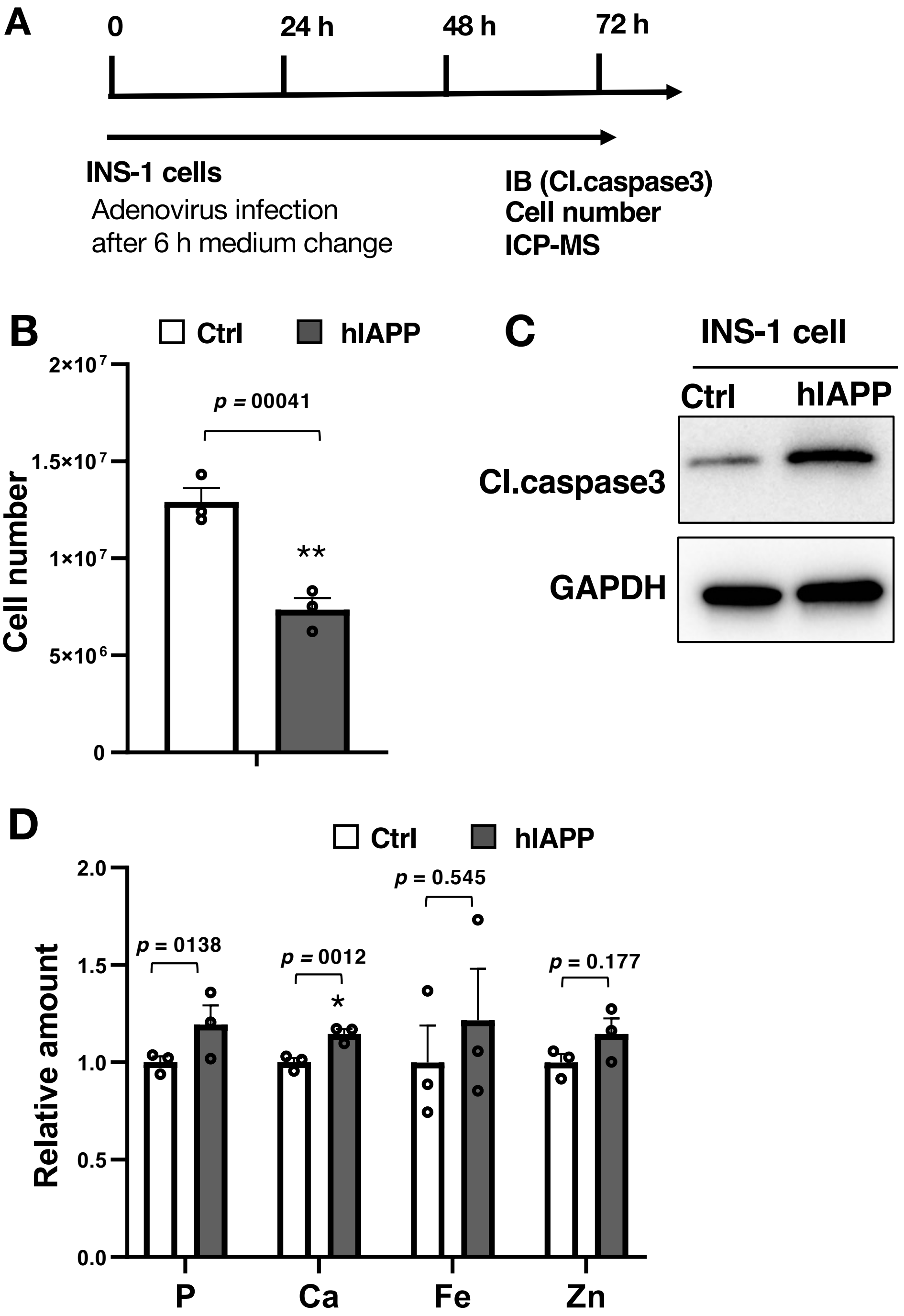

Fig. S5. Expression of hIAPP in INS-1 cells does not result in significant changes in metal levels.

(A) Schematic representation of the experimental design. INS-1 cells were infected with adenoviruses expressing LacZ and hIAPP and metal contents were measured by ICP-MS after 72 h of adenovirus infection. (B) The number of cells not stained with Trypan Blue was counted. (C) Western blotting of the indicated proteins in INS-1 cells after the infection of an adenovirus expressing LacZ (Ctrl) or hIAPP. (D) Relative amounts of each element in indicated cells were measured using ICP-MS. Data are shown as means ± SEM. *$p < 0.05$, **$p < 0.01$(Ctrl vs hIAPP).
